# Rola Emolientów w Atopowym Zapaleniu Skóry u Dzieci

**DOI:** 10.34763/devperiodmed.20182204.396403

**Published:** 2019-01-14

**Authors:** Ewa Kamińska

**Affiliations:** 1 Zakład Farmakologii, Instytut Matki i Dziecka, Warszawa, Polska

**Keywords:** atopowe zapalenie skóry, dzieci, emolienty, humektanty, badania kliniczne, atopic dermatitis, children, emollients, humectants, clinical trials

## Abstract

Atopowe zapalenie skóry jest przewlekłą chorobą zapalną, przebiegającą z nawrotowymi zaostrzeniami, uporczywym świądem, rumieniem, suchością skóry wskutek uszkodzenia bariery naskórkowej i zakażeniami gronkowcowymi. Czynnikami wywołującymi są mutacje w genie kodującym filagrynę, rozregulowanie układu immunologicznego, zmiany dotyczące mikrobiomu skóry i lipidów w stratum corneum, niedobór peptydów antymikrobiologicznych AMPs. Choroba dotyczy głównie dzieci, powodując znaczne pogorszenie jakości życia, a jej pierwsze objawy występują w około 90% przypadków przed ukończeniem 5. r.ż. Przez lata termin „emolienty” odnoszono do substancji natłuszczających stosowanych w celu uelastycznienia i zmiękczenia skóry w chorobach przebiegających z szorstkością, złuszczaniem i suchością skóry. Obecnie wiadomo, że emolienty mogą także działać nawilżająco poprzez zatrzymywanie wody w skórze, stąd często terminy „emolient” i „substancja nawilżająca” są używane zamiennie. Zgodnie z najnowszymi rekomendacjami towarzystw dermatologicznych podstawową terapią w atopowym zapaleniu skóry jest długotrwałe stosowanie preparatów emoliencyjnych aplikowanych regularnie na skórę oraz dodawanych do kąpieli. Emolienty mogą być stosowane w monoterapii lub –w okresach zaostrzeń –w skojarzeniu z miejscowo stosowanymi kortykosteroidami lub inhibitorami kalcyneuryny. Badania kliniczne wykazały, że systematyczne stosowanie preparatów emoliencyjnych pomaga zarówno przywrócić funkcje ochronne bariery skórnej, jak i wpływa na zmniejszenie zużycia miejscowo stosowanych kortykosteroidów u niemowląt, dzieci i dorosłych pacjentów z atopowym zapaleniem skory. Wyniki badań oraz wieloletnie doświadczenie kliniczne dowodzą skuteczności i bezpieczeństwa stosowania preparatów emoliencyjnych. W pracy przedstawiono aktualny stan wiedzy na temat emolientów, tj. przegląd składników preparatów emoliencyjnych, właściwości i mechanizmy działania emolientów, a także omówiono ich znaczenie w atopowym zapaleniu skóry oraz wyniki badań klinicznych przeprowadzonych z udziałem dzieci z atopowym zapaleniem skóry.

## Abstract (pl)

Atopic dermatitis is a chronic inflammatory disease characterized by recurrent flares, intense itching, erythema, dry skin resulting from skin barrier defects, and staphylococcal infections. Multiple factors may affect the skin`s normal barrier function, including filaggrin gene mutations, immune dysregulation, altered skin microbiome, altered lipids in stratum corneum, or deficiency of antimicrobial peptides AMPs. The disease mainly affects children, causing a considerable impact on the quality of their life; its first manifestations occur with up to 90% of cases before the age of 5. For years emollients have been known as oily substances used to treat rough, scaling, xerotic conditions to make skin flexible and soft. Recently, we have learned that emollients can also moisten and hydrate dry skin, so the terms „emollient” and „moisturizer” are often used interchangeably. According to current management guidelines on atopic dermatitis prepared by dermatological societies, long-term emollient application direct to the skin and as bath additives are the basic therapy of atopic dermatitis. Emollients may be used in monotherapy or – in the flares – in conjunction with topical corticosteroids or calcineurin inhibitors. Clinical trials proved that regular emollient application moistens and hydrates the skin and helps the skin maintain a defensive barrier effect as well as reduces the amount of topical corticosteroids needed for atopic eczema in infants, children and adult patients. The results of trials and long clinical experience proved that emollients are safe and effective in patients with atopic dermatitis. This paper presents information based on recent knowledge concerning emollients: an overview of emollient components, their properties, mechanism of action, and the role they play in atopic eczema, as well as the results of clinical trials performed in children with atopic dermatitis.

## Wprowadzenie

Przez dziesiątki lat określenie „emolient” dotyczyło wyłącznie substancji będącej składnikiem produktu kosmetycznego, której zadaniem było natłuszczenie, zmiękczenie, wygładzenie i pośrednie nawilżenie skóry, i takie nazewnictwo zastosowano w niniejszej pracy. Obecnie w terminologii kosmetologicznej i dermatologicznej spotykamy się coraz częściej z tym określeniem w odniesieniu do gotowego produktu kosmetycznego o właściwościach nawilżających i natłuszczających, w skład którego wchodzi jeden lub kilka emolientów. Ponieważ jednak taki kosmetyk zawiera nie tylko emolienty, ale też inne składniki recepturowe, w niniejszej pracy nosi on nazwę produktu emoliencyjnego. Z punktu widzenia idealnego działania takiego kosmetyku najlepiej jest, jeśli zawiera on emolienty w połączeniu z substancjami wiążącymi i zatrzymującymi wodę w skórze, czyli tzw. humektantami. Dzięki temu składniki lipofilowe (emolienty) i hydrofilowe (humektanty) uzupełniają swoje działanie, zapobiegając suchości skóry.

## Właściwości emolientów

Emolienty są cenionymi składnikami kosmetyków dla niemowląt, dzieci i osób dorosłych, zwłaszcza ze skórą atopową. Są stosowane zarówno w kosmetykach pozostających na skórze (m.in. kremy, balsamy, mleczka, emulsje), jak i w preparatach myjących (emulsje i olejki do kąpieli, szampony, żele pod prysznic). Ich nazwa pochodzi od łacińskiego „*emolliere*”, tzn. zmiękczać. Są jednymi z najstarszych składników kosmetyków pielęgnacyjnych. Oleje roślinne o właściwościach emoliencyjnych stosowano już w starożytnym Egipcie i Chinach, lanolinę – w starożytnej Grecji, a historia wazeliny sięga drugiej połowy XIX w. Obecnie emolienty przeżywają „drugą młodość”, a ich stosowanie nie jest już tylko intuicyjne, lecz także poparte wynikami badań. Substancje te znalazły zastosowanie zarówno w leczeniu, jak i profilaktyce wielu chorób skóry, zwłaszcza w atopowym zapaleniu skóry (AZS), łuszczycy, wyprysku kontaktowym. Stosowanie preparatów emoliencyjnych ogranicza często konieczność leczenia glikokortykosteroidami i inhibitorami kalcyneuryny, wydłuża okres remisji objawów, a niekiedy nawet zapobiega nawrotom choroby [1].

Większość emolientów wykazuje działanie epidermalne, gdyż nie przenikają one przez naskórek, lecz tworzą na jego powierzchni cienki film − barierę okluzyjną − zabezpieczającą przed nadmiernym odparowywaniem wody z głębszych warstw skóry. Inne, jak np. lanolina i ceramidy, penetrują do struktur cementu międzykomórkowego warstwy rogowej naskórka, trwale odbudowując barierę naskórkową [2]. Stopień nawilżenia skóry zależy od właściwości higroskopijnych warstwy rogowej naskórka i jej zdolności barierowych umożliwiających zatrzymanie wody, na co mają wpływ lipidy powierzchniowe, czyli tzw. płaszcz lipidowy. Ważnym parametrem umożliwiającym ocenę stopnia uszkodzenia bariery naskórkowej jest wartość TEWL (*Transepidermal Water Loss*), określająca przeznaskórkową utratę wody. W wyniku utworzenia okluzji emolienty zatrzymują wodę w naskórku, powodując zmniejszenie TEWL [3], dzięki czemu wywierają pośrednie działanie nawilżające. Efektem działania emolientów jest rozluźnienie, uelastycznienie i zwiększenie hydratacji warstwy keratynocytów − komórek naskórka odpowiedzialnych za syntezę keratyny, lipidów i naturalnego czynnika nawilżającego NMF (*Natural Moisturizing Factor*). W skład NMF wchodzą substancje o silnym działaniu nawilżającym, głównie aminokwasy i ich pochodne, kwas piroglutaminowy (PCA), kwas urokainowy, sole i jony nieorganiczne, kwas mlekowy, mocznik. Naturalny czynnik nawilżający, który stanowi 15-30% masy warstwy rogowej naskórka, wpływa regulująco na wiązanie wody w skórze, a jego zmniejszenie o ok. 10% powoduje znaczącą suchość skóry. Stosowanie emolientów prowadzi do wyraźnej poprawy uwodnienia warstwy rogowej naskórka oraz zmiękczenia i wygładzenia skóry. Ponadto, dzięki zdolności odtwarzania lipidów naskórka wymytych przez substancje powierzchniowo czynne zawarte w preparatach myjących, emolienty wpływają na regenerację naskórka i przywracają jego prawidłową funkcję. Poprawa stanu nawilżenia i natłuszczenia skóry wpływa na jej elastyczność, a zdolność do regeneracji naskórka zabezpiecza przed uszkodzeniem, pęknięciami i nadmiernym łuszczeniem oraz wnikaniem niepożądanych substancji egzogennych. Długotrwałe i systematyczne stosowanie kosmetyków emoliencyjnych zmniejsza odczucie świądu skóry, który jest jednym z najbardziej dokuczliwych objawów AZS, zmniejsza także stan zapalny skóry.

Ze względu na różnorodność emolientów, odmienne mechanizmy ich działania nawilżającego, regeneracyjnego, przeciwświądowego oraz zdolności poprawy zaburzonej charakterystyki mikrobiomu skóry u chorych na AZS, istotny jest odpowiedni, indywidualny dobór preparatu emoliencyjnego w zależności od stanu klinicznego pacjenta i oczekiwanego efektu terapeutycznego.

## Przegląd emolientów

Grupa emolientów obejmuje substancje naturalne oraz otrzymywane syntetycznie. W piśmiennictwie brak jest jednoznacznej klasyfikacji emolientów. Występuje duża różnorodność podziałów zarówno pod względem budowy chemicznej, właściwości, jak i źródła otrzymywania tych substancji. Nie ułatwia też sprawy fakt, że w niektórych opracowaniach anglojęzycznych termin „moisturisers” (preparaty o działaniu nawilżającym) jest stosowany zamiennie z terminem „emollients” (preparaty emoliencyjne) [4, 5].

Przedstawiony poniżej podział emolientów stanowi próbę połączenia i uproszczenia różnych systemów klasyfikacji tych związków.

### Estry, triglicerydy, alkohole tłuszczowe, kwasy tłuszczowe

W tej grupie ważne miejsce zajmują syntetyczne estry kwasów tłuszczowych, zwłaszcza mirystynian izopropylu i mirystynian mirycylu, oraz otrzymywany z oleju palmowego palmitynian izopropylu. Nie tylko natłuszczają i zmiękczają naskórek, ale także umożliwiają wymianę tlenową i wodną, nie pozostawiając uczucia lepkości. Są często stosowane jako dodatek do olejów mineralnych, gdyż powodują częściowe otwarcie warstwy okluzyjnej powstałej po zastosowaniu parafin, dzięki czemu umożliwiają wymianę między skórą i otoczeniem. Cennym składnikiem pochodzenia naturalnego jest trigliceryd kaprylowo-kaprynowy (*Caprylic/Capric Trigliceryde*). Właściwości emoliencyjne wykazują także inne związki z tej grupy, m.in. alkohol cetylowy i kwas stearynowy.

### Ceramidy i cholesterol

Ceramidy i cholesterol są związkami lipidowymi, naturalnymi składnikami cementu międzykomórkowego w warstwie rogowej naskórka. Stanowią nieprzepuszczalną dla wody warstwę i decydują o elastyczności skóry. Cholesterol jest od lat szeroko stosowany w postaci popularnej maści cholesterolowej (często w połączeniu z witaminami A i E), znanej z dużej skuteczności u pacjentów z AZS, a także jest popularnym podłożem wielu maści.

Zawartość ceramidów w skórze zmniejsza się z wiekiem, są też wypłukiwane przez środki myjące, co zaburza funkcje ochronne skóry i powoduje zwiększenie TEWL. Ceramidy są często stosowane w połączeniu z innymi składnikami naturalnego płaszcza lipidowego: cholesterolem, kwasami tłuszczowymi, skwalenem, fosfolipidami, co przyczynia się do odbudowy bariery skórno-naskórkowej i nawilżenia skóry. Ich skuteczność w postaci zwiększenia uwodnienia skóry i zmniejszenia TEWL oraz odnowy bariery naskórkowej została potwierdzona w badaniach [6]. Dzięki ich stosowaniu ograniczeniu ulega przenikanie przez skórę substancji egzogennych, w tym alergenów, oraz zmniejsza się świąd.

### Oleje roślinne

Oleje roślinne ze względu na swe właściwości pielęgnacyjne są często stosowane w kosmetykach dla niemowląt i dzieci. Są bardzo cenionymi surowcami ze względu na zawartość nienasyconych kwasów tłuszczowych i innych składników wpływających na odbudowę lipidów naskórka, m.in. poprzez udział w tworzeniu cementu międzykomórkowego [7]. Najczęściej stosowane w kosmetykach przeznaczonych dla dzieci ze skórą atopową są bogate w kwas linolowy oleje ze słodkich migdałów (*Prunus dulcis*) i słonecznika (*Helianthus annuus*) oraz zawierające cenny kwas gamma-linolenowy oleje wiesiołkowy (*Oenothera biennis* i *O. paradoxa*) i ogórecznikowy (*Borago officinalis*). Bardzo korzystne właściwości mają też: olej z oliwek, czyli bogata w kwas oleinowy oliwa, olej z pestek winogron (*Vitis vinifera*), kiełków pszenicy (*Triticum vulgare Germ Oil*), masłosza (*Butyrospermum parkii*, czyli masło shea), orzechów makadamii (*Macadamia ternifolia*), awokado (*Persea gratissima*), jojoby (*Simmondsia chinensis*), nasion lnu (*Linum usitatissimum*). Dzięki korzystnemu działaniu na skórę oleje te są stosowane także w kosmetykach dla dzieci ze skórą atopową. Ponadto cennym surowcem roślinnym jest owies (*Avena sativa*); uzyskany z niego ekstrakt − mleczko owsiane − ma działanie łagodzące i przeciwzapalne [7]. Należy pamiętać, że dzieci z alergią pokarmową na soję lub orzeszki arachidowe mogą reagować objawami alergii skórnej na olej sojowy i arachidowy wchodzące w skład kosmetyków.

Nową, cenną grupą emolientów są polihydroksylowane oleje roślinne, które charakteryzują się korzystniejszymi właściwościami fizyko-chemicznymi, tj. lepszą rozprowadzalnością, mniejszą kleistością itp., przy zachowanym bardzo dobrym działaniu nawilżającym skórę.

### Woski i oleje odzwierzęce

Lanolina (wosk owczy) jest emolientem otrzymywanym z wełny owczej, regulującym gospodarkę wodną skóry i zmiękczającym naskórek. Jest szeroko stosowana w kosmetyce i farmacji ze względu na zdolność znacznego wiązania wody. Nawilżająco działają jej składniki, głównie sterole i ich estry oraz wolne i zestryfikowane kwasy tłuszczowe, które penetrują do struktur cementu międzykomórkowego. Lanolina i jej pochodne należą do emolientów trwale odbudowujących barierę naskórkową, gdyż efekty ich działania utrzymują się dłużej niż w przypadku emolientów okluzyjnych [2]. Struktura lipidów lanolinowych jest podobna do struktury lipidów mazi płodowej (*vernix caseosa*), co tłumaczy bardzo korzystne działanie lanoliny na barierę naskórkową noworodków [8]. W przeszłości lanolina była stosunkowo często przyczyną alergii, za którą odpowiada frakcja wolnych alkoholi lanolinowych. Podobnie jak w przypadku parabenów, reakcje alergiczne na lanolinę, a także na eucerynę, występowały przede wszystkim u pacjentów ze zmianami skórnymi, głównie owrzodzeniem ud („paradoks lanolinowy”). Obecnie dysponujemy już niealergizującymi pochodnymi (lanolina ciekła, acetylowane i oksyetylenowane alkohole lanolinowe), które charakteryzują się także lepszymi właściwościami fizyko-chemicznymi.

Właściwości emoliencyjne wykazuje też wosk pszczeli (*Cera alba, Cera flava, Beeswax*). Cennym surowcem kosmetycznym jest skwalen (*Squalene)* - biogenny emolient o dużym powinowactwie do skóry, składnik płaszcza lipidowego. Tworzy na powierzchni skóry film okluzyjny, pełniący rolę bariery ochronnej.

### Oleje mineralne (parafiny)

Parafiny są grupą surowców obejmującą m.in. stosowaną od 1872 r. wazelinę (*Petrolatum*), olej parafinowy (olej mineralny, *Paraffinum liquidum*), cerezynę, ozokeryt, wosk mikrokrystaliczny (*Cera microcristallina*). Ich właściwości fizyczne są zbliżone do właściwości tłuszczów roślinnych i zwierzęcych, jednak w odróżnieniu od nich charakteryzują się doskonałą stabilnością, m.in. nie ulegają rozkładowi pod wpływem UV i tlenu. Wywierają silne działanie okluzyjne, tworząc na powierzchni skóry warstwę lipidową, dzięki czemu działają zmiękczająco, natłuszczająco i pośrednio nawilżająco, gdyż zatrzymują wodę w skórze. Jednym z najczęściej stosowanych emolientów jest wazelina, która nie tylko nawilża skórę, ale także wpływa na odbudowę kwaśnego płaszcza lipidowego pełniącego rolę bariery ochronnej skóry [5]. Najnowsze badania wazeliny wykazały, że mechanizm jej działania jest bardziej skomplikowany niż dotychczas sądzono. Emolient działa na poziomie molekularnym, indukując m.in. aktywność peptydów antymikrobiologicznych (AMPs − *antimicrobial peptides*), a tym samym przyczynia się do zwalczania patogenów bakteryjnych, w tym *Staphylococcus aureus* [9].

Parafiny są mieszaniną alkanów otrzymywanych w procesie destylacji określonej frakcji ropy naftowej o wysokim stopniu oczyszczenia, przede wszystkim z pozostałości benzo-α-pirenu i dimetylosulfotlenku, których dotyczą zaostrzone normy jakościowe. Z tego względu w kosmetykach stosowana jest np. wazelina biała farmaceutyczna o wysokim stopniu oczyszczenia, która nie jest zaliczana do substancji niebezpiecznych. Bezpieczeństwo stosowania parafin zostało potwierdzone przez liczne agendy, w tym zespół ekspertów WHO, Cosmetics Ingredients Review i komitet naukowy Europejskiego Urzędu ds. Bezpieczeństwa Żywności (EFSA) [10]. Wbrew internetowym wpisom przeciwników tej grupy związków, są one bezpieczne w kosmetykach nawet po zastosowaniu w dużym stężeniu, a niewielka domieszka innych substancji tłuszczowych, np. estrów syntetycznych (mirystynian izopropylu) lub silikonu zapobiega wystąpieniu zaburzeń funkcji barierowej skóry. Dzięki właściwościom hydrofobowym parafiny nie przenikają przez *stratum corneum* i żywe warstwy naskórka, ani do skóry właściwej i nie kumulują się w organizmie. Nie wykazano także, aby wazelina i olej parafinowy działały komedogennie, aczkolwiek nie zaleca się ich stosowania w cerze trądzikowej. Oleje mineralne znalazły szerokie zastosowanie nie tylko w wyrobach kosmetycznych, ale także − jako substancje o wieloletnim, ugruntowanym zastosowaniu medycznym − są bazą dla większości maści, czopków i innych leków stosowanych na skórę zmienioną chorobowo.

### Oleje silikonowe (polidimetylosiloksany, PDMS)

Ich inna nazwa to polimery filmotwórcze, gdyż tworzą na powierzchni skóry niewidoczny i odporny na zmywanie film okluzyjny, który jednak nie zaburza jej funkcji. Najpopularniejszymi substancjami z tej grupy są polidimetylosiloksan (dimetykon), cyklopolidimetylosiloksan (cyklometykon), cyklopentasiloksan, cykloheksasiloksan. Podobnie jak parafiny, silikony są obojętne chemicznie i wyjątkowo stabilne, tj. odporne na działanie UV, tlenu i wody. Ich niewielka ilość dodana do kosmetyku emoliencyjnego zawierającego olej parafinowy znacznie poprawia jego właściwości sensoryczne.

## Humektanty

Jak już wspomniano, humektanty są substancjami o właściwościach higroskopijnych, które wykazują zdolność wiązania i zatrzymania wody w warstwie rogowej naskórka. Do grupy tej należą m.in. gliceryna, mocznik, kwas hialuronowy, glikole, aminokwasy, kwas mlekowy i mleczan sodu, alantoina, pantenol. Niektóre z nich wchodzą w skład naturalnego czynnika nawilżającego NMF. Odpowiednie nawodnienie warstwy rogowej zapewnia właściwą elastyczność skóry, chroni ją przed uszkodzeniami oraz zapewnia utrzymanie homeostazy skóry.

Gliceryna jest popularnym składnikiem preparatów emoliencyjnych, działającym nie tylko nawilżająco, ale także ułatwiającym wchłanianie innych substancji.

Mocznik w stężeniu do 10% wywiera działanie nawilżające, co jest szczególnie korzystne u dzieci z atopowym zapaleniem skóry. W piśmiennictwie znajdują się jednak doniesienia o zwiększonym, toksycznym stężeniu mocznika we krwi u noworodków i niemowląt po zastosowaniu preparatów zawierających mocznik [11]. Związek ten należy więc stosować z zachowaniem dużej ostrożności u małych dzieci. Zgodnie z rekomendacjami Niemieckiego Towarzystwa Dermatologicznego (DDG) [12] mocznika nie należy stosować u niemowląt, a niektórzy autorzy są nawet zdania, że nie powinno się go stosować u dzieci do 6. r.ż. [13]. Mocznik, który w stężeniach powyżej 10% działa złuszczająco, a w stężeniach 30-50% − keratolitycznie, może wywierać działania niepożądane. U dzieci poniżej 6. rż. lepiej tolerowane są preparaty z gliceryną niż mocznikiem [14].

Ze względu na silne działanie nawilżające kwasu hialuronowego jego stosowanie wpływa na poprawę uwodnienia *stratum corneum* z jednoczesnym zmniejszeniem TEWL, dzięki czemu zmniejsza się suchość i świąd skóry. Utworzenie ochronnego filmu na powierzchni skórnych zmian atopowych i korzystny wpływ w postaci przyspieszenia gojenia zadrapań i zmian wypryskowych oraz regeneracji uszkodzonej skóry, wpływają na odtworzenie prawidłowej bariery naskórkowej [15]. Podkreśla się rolę kwasu hialuronowego, podobnie jak innych preparatów emoliencyjnych, w leczeniu i proftlaktyce zarówno AZS, jak i innych chorób przebiegających z nadmierną suchością (*xerosis*), złuszczaniem, świądem, uszkodzeniem i (lub) podrażnieniem skóry.

Aminokwasy, kwas mlekowy i mleczan sodu są składnikami NMF i zapewniają skuteczne nawilżenie warstwy rogowej naskórka. Pantenol silnie nawilża i zmiękcza skórę, wpływa także aktywizująco na podziały komórkowe, pobudzając wzrost oraz odnowę komórek naskórka i skóry właściwej. Działa regenerująco, łagodzi podrażnienia (w tym powstałe w wyniku oparzeń słonecznych) i przyspiesza gojenie uszkodzeń skóry [16]. Podobne działanie ma alantoina, która ponadto wykazuje właściwości zmiękczania tkanki łącznej.

## Atopowe zapalenie skóry a zaburzenia mikrobiomu

Atopowe zapalenie skóry jest chorobą o złożonej etiopatogenezie, charakteryzującą się nieprawidłową budową i działaniem warstwy rogowej naskórka. Jest najczęściej występującą zapalną chorobą skóry u dzieci. Jej pierwsze objawy w około 60% przypadków manifestują się w okresie niemowlęcym, a w około 90% przypadków − przed ukończeniem piątego roku życia [4]. Zachorowalność na AZS stale się zwiększa. W 2000 r. szacowano, że w krajach wysoko rozwiniętych obejmuje ona 15-20% populacji dzieci [17], natomiast van Zuuren i wsp. (2017) podają, że wynosi ona do 30% u dzieci i 2-10% u dorosłych [4]. Suchość skóry i charakterystyczne dla AZS zmiany skórne, a przede wszystkim uporczywy świąd powodujący zaburzenia snu i – zwłaszcza u dzieci − drapanie się, prowadzące do bakteryjnych nadkażeń skóry, powodują znaczące pogorszenie jakości życia chorych na AZS [1].

Głównym czynnikiem wywołującym AZS są mutacje w genie kodującym filagrynę, która jest białkiem odpowiadającym za prawidłową funkcję bariery naskórkowej [18]. Mutacje te powodują zaburzenia procesu dojrzewania keratynocytów, a w ich następstwie uszkodzenie bariery naskórkowej. Jego efektem jest upośledzenie nawilżenia skóry wskutek nadmiernej przeznaskórkowej utraty wody (TEWL) i zmniejszenia zawartości naturalnego czynnika nawilżającego (NMF) w *stratum corneum* [19, 20], a także większa podatność na działanie czynników drażniących i nasilone ryzyko przenikania ksenobiotyków przez uszkodzony naskórek, w tym alergenów, drobnoustrojów i toksyn [4]. Wśród czynników wywołujących AZS wymienia się też rozregulowanie układu immunologicznego, zmiany w obrębie mikrobiomu skóry, zaburzenia składu lipidów w *stratum corneum*, niedobór peptydów antymikrobiologicznych (AMPs) [21].

Zmiany w mikrobiomie, czyli zaburzenia równowagi mikroflory bakteryjnej zasiedlającej skórę, odgrywają znaczącą rolę w etiopatogenezie AZS. Na skórze pacjentów z ciężkim AZS dominuje *S. aureus*, bakteria kolonizująca od 60 do 100% pacjentów [4] z tą chorobą, podczas gdy w lżejszych postaciach choroby występuje przewaga *S. epidermidis* [21]. W mikrobiomie skóry niemowląt w wieku 12 mies.ż. ze zdiagnozowanym AZS także zdecydowanie przeważają bakterie *S. aureus* nad gronkowcami komensalnymi, co sugeruje, że bakterie komensalne mogą chronić przed rozwojem AZS [22]. Bakterie komensalne indukują AMPs, hamując rozwój patogenów, natomiast w przypadku dominacji tych ostatnich dochodzi do zaburzeń odpowiedzi immunologicznej w skórze, rozwoju stanu zapalnego i defektu bariery naskórkowej [21]. Istotną rolę w jej uszkodzeniu odgrywają proteazy uwalniane przez *S. aureus* [19, 23]. Wytwarzane przez nie toksyny zaostrzają stan zapalny i pogarszają przebieg choroby [23]. W okresie poprzedzającym zaostrzenie stanu zapalnego występuje wyraźna dominacja *S. aureus* nad innymi szczepami, co zaburza prawidłowe funkcjonowanie bariery skórnej. Ostatnio wykazano, że u niemowląt kolonizacja skóry przez *S. aureus* koreluje dodatnio z wystąpieniem AZS w późniejszym okresie niemowlęcym oraz że nasilenie kolonizacji poprzedza o 2 miesiące wystąpienie pierwszych objawów AZS [24].

## Badania kliniczne emolientów u pacjentów z AZS

Przed 2000 r. przeprowadzono pięć [17], a po tym czasie co najmniej 20 badań, których celem była ocena skuteczności i bezpieczeństwa stosowania preparatów emoliencyjnych u dorosłych i dzieci, w tym także u noworodków i niemowląt. Nie wszystkie badania przeprowadzono z randomizacją. Większość z nich obejmowała pacjentów z łagodnym lub umiarkowanym AZS, u których preparaty te stosowano 2-3 razy dziennie, porównując ich skuteczność z placebo, innym preparatem zawierającym emolienty lub miejscowo stosowanym glikokortykosteroidem. Średni czas stosowania preparatu emoliencyjnego wynosił 6,7 tygodnia [4]. W niektórych badaniach dopuszczano stosowanie steroidów zarówno w grupie badanej, jak i kontrolnej, dzięki czemu możliwe było ustalenie czy preparaty emoliencyjne mają wpływ na zmniejszenie stosowania steroidów.

Wyniki większości badań wykazały, że stosowanie u dorosłych pacjentów z AZS preparatów emoliencyjnych wzmacnia barierę naskórkową i znacznie poprawia stan skóry, wpływając na zmniejszenie suchości, szorstkości i złuszczania skóry oraz odczucia świądu, a nawet prowadzi do istotnego wydłużenia fazy regresji objawów [25,26,27,28,29,30,31,32]. Zmniejsza także potrzebę miejscowego stosowania glikokortykosteroidów u tych pacjentów, u których ich skuteczność była taka sama jak preparatów emoliencyjnych, pozwalając tym samym na uniknięcie działań niepożądanych wywołanych przez preparaty steroidowe [32, 33, 34, 35, 36, 37]. Niektóre badania wykazały, że jednoczesne stosowanie preparatów emoliencyjnych wpływa na zwiększenie skuteczności kortykosteroidów [25, 38].

Podobne wyniki uzyskano w badaniach przeprowadzonych u niemowląt i dzieci [35, 36, 37, 38]. W dwóch badaniach z udziałem łącznie 166 dzieci w wieku od 5. mies. życia do 5. r.ż., w których porównywano przez 3 tygodnie skuteczność preparatu emoliencyjnego (pochodna olejowa otrzymana ze słonecznika) oraz kremów zawierających kortykosteroidy (0,1% maślanopropionian hydrokortyzonu lub 0,05% dezonid), skuteczność preparatu emoliencyjnego była podobna do skuteczności każdego z kremów steroidowych, co pozwoliło na zmniejszenie stosowania tych ostatnich [36, 37]. W randomizowanym badaniu [35], przeprowadzonym z udziałem 173 niemowląt z umiarkowanym lub ciężkim AZS, stosowanie 2 razy dziennie preparatu emoliencyjnego w postaci mleczka znacząco zmniejszyło w ciągu 6 tygodni zużycie kremu steroidowego (0,1% dezonid) w porównaniu z grupą kontrolną, w której nie stosowano preparatu emoliencyjnego (42% *vs* 7,5%; p>0,05). Simpson i wsp. (2011) w badaniu z udziałem 127 pacjentów w wieku ≥ 3 lat odnotowali szybsze ustępowanie objawów chorobowych w 7., 14. i 21. dniu stosowania 2 razy dziennie preparatu emoliencyjnego (zawierającego ceramidy, emolienty, humektanty) jednocześnie z preparatem steroidowym, niż po stosowaniu samego steroidu (p<0,005) [38].

Stosowanie preparatów emoliencyjnych u niemowląt i osób dorosłych z AZS zmniejsza o 50-78% kolonizację *S. aureus*, z jednoczesnym obniżeniem pH skóry i zwiększeniem liczby szczepów *Streptococcus salivarius*, bakterii wykazujących właściwości immunomodulacyjne [39]. Najnowsze badania wykazały, że profilaktyczne stosowanie preparatów emoliencyjnych u noworodków obciążonych ryzykiem wystąpienia AZS zmniejsza to ryzyko nawet o 50%, nie tylko wskutek regeneracji bariery naskórkowej i zmniejszenia TEWL, ale przede wszystkim w wyniku zmian w mikrobiomie skóry i statystycznie istotnego obniżenia jej pH [39, 40]. Niektórzy autorzy sugerują, że wczesne wykrycie u noworodka zaburzeń mikroflory bakteryjnej skóry z jednoczesnym zwiększeniem TEWL może zapobiec rozwojowi AZS, pod warunkiem korekty dysbiozy i szybkiego rozpoczęcia stosowania preparatów emoliencyjnych [21]. Wykazano także, że u noworodków przedwcześnie urodzonych stosowanie preparatów emoliencyjnych zmniejsza ryzyko wystąpienia zakażeń skóry [42].

Na uwagę zasługują wyniki randomizowanych badań skuteczności emoliencyjnego wyrobu medycznego z aptecznej grupy tzw. PEDs (*prescription emollient devices*), jakim jest Atopiclar (MAS063DP), przeprowadzone zarówno u dorosłych, jak i u dzieci. Preparaty te oprócz składników hydrofilowych i lipofilowych zawierają także związki działające przeciwzapalnie. Atopiclar jest hydrolipidowym kremem emoliencyjnym, który oprócz substancji nawilżających i odbudowujących barierę naskórkową (masło shea, kwas hialuronowy, estry kwasów tłuszczowych) zawiera związki o działaniu przeciwzapalnym i przeciwświądowym (kwas gliceretynowy z korzenia lukrecji, ekstrakt z pestek winogron *Vitis vinifera*, telmesteina). Wyniki 4 wieloośrodkowych badań randomizowanych [43, 44, 45, 46] z udziałem 450 pacjentów z AZS (w tym 202 niemowląt i dzieci) wykazały znaczące zmniejszenie świądu i zmian wypryskowych u pacjentów stosujących Atopiclar 3 razy dziennie przez 21-50 dni, w porównaniu z grupą kontrolną, u której stosowano podłoże kremu. W przeprowadzonym przez Millera i wsp. w 2011 r. [47] nierandomizowanym badaniu z udziałem 39 dzieci w wieku 3.-17. r.ż. nie wykazano przewagi preparatu Atopiclar nad kremem barierowym zawierającym ceramidy, kwasy tłuszczowe i cholesterol, ani nad preparatem emoliencyjnym na bazie wazeliny. Autorzy podkreślają natomiast, że koszt preparatu emoliencyjnego z wazeliną był 47 razy mniejszy niż pozostałych schematów leczenia.

We wszystkich opisanych badaniach zarówno u dzieci, jak i u dorosłych, odnotowano jedynie nieliczne działania niepożądane, odnoszące się zazwyczaj do preparatów zawierających większe stężenia mocznika.

## Rekomendacje towarzystw dermatologicznych

Przedstawione powyżej wyniki badań skuteczności i bezpieczeństwa stosowania preparatów emoliencyjnych stały się podstawą do włączenia tych preparatów do pierwszej linii postępowania u dzieci i pacjentów dorosłych z atopowym zapaleniem skóry. W łagodnym AZS mogą być stosowane jako jedyne postępowanie, w okresach zaostrzeń – o ile zachodzi taka potrzeba - można je łączyć z kortykosteroidami lub inhibitorami kalcyneuryny. Rekomendacje te obowiązują w Stanach Zjednoczonych [48] i krajach europejskich [7, 12, 14, 49]. Zasady pielęgnacji zarówno w okresie remisji choroby, jak i jej zaostrzeń kładą nacisk na rolę systematycznego nawilżania i natłuszczania skóry za pomocą preparatów emoliencyjnych, stosowanych do mycia oraz aplikowanych kilkakrotnie w ciągu dnia (minimum 2 razy dziennie) na skórę całego ciała. Zaleca się stosować preparaty, które nie zawierają składników o działaniu alergizującym, a więc przede wszystkim kompozycji zapachowych i konserwantów. Ważny jest także indywidualny dobór preparatu emoliencyjnego, np. w przypadku znacznego nasilenia suchości skóry lepsza będzie bardziej tłusta emulsja typu W/O (woda/olej), a w stanach podostrych – O/W (olej/woda). Na skuteczność preparatu wpływa też stosowanie odpowiedniej jego ilości. Prawidłowe zużycie preparatu emoliencyjnego u dzieci powinno wynosić 150-200 g na tydzień, a u dorosłych – 500 g na tydzień [7].

## Podsumowanie

Emolienty są składnikami preparatów emoliencyjnych − kosmetyków i wyrobów medycznych, wykazujących działanie nawilżające, natłuszczające i zmiękczające skórę. W preparatach tych emolienty są często stosowane w połączeniu z humektantami, dzięki czemu oba składniki nasilają swoje działanie nawilżające.

Ze względu na wywierane działanie nawilżające i zdolność odbudowy uszkodzonej bariery naskórkowej preparaty emoliencyjne były od lat stosowane tradycyjnie u pacjentów z suchością skóry.

Przeprowadzone w ostatniej dekadzie badania z udziałem dzieci i dorosłych pacjentów potwierdziły skuteczność i bezpieczeństwo stosowania preparatów emoliencyjnych w atopowym zapaleniu skóry. Badania te wykazały także, że systematyczne stosowanie preparatów emoliencyjnych zmniejsza zapotrzebowanie na miejscowo stosowane glikokortykosteroidy, a tym samym przyczynia się do ograniczenia działań niepożądanych wywoływanych przez te leki.

Najnowsze badania wykazały, że profilaktyczne stosowanie preparatów emoliencyjnych u noworodków obciążonych ryzykiem wystąpienia AZS zmniejsza to ryzyko nawet o 50%, nie tylko wskutek regeneracji bariery naskórkowej i zmniejszenia TEWL, ale przede wszystkim w wyniku zmian w mikrobiomie skóry i obniżenia jej pH.

Na podstawie wyników badań opracowano w wielu krajach rekomendacje, zgodnie z którymi stosowanie preparatów emoliencyjnych zostało włączone do pierwszej linii postępowania w atopowym zapaleniu skóry u dzieci i dorosłych pacjentów. W zależności od przebiegu choroby mogą one być aplikowane w monoterapii lub w połączeniu z miejscowo stosowanymi glikokortykosteroidami (terapia przerywana) bądź inhibitorami kalcyneuryny (terapia proaktywna).

## References

[1] Nankervis H, Thomas KS, Delamere FM i wsp. (2016). Scoping systematic review of treatments for eczema. Programme Grants Appl Res.

[2] Pytkowska K, Głowacka K, Arct J (2014). Lanolina i surowce z niej otrzymywane we współczesnej kosmetologii. Świat Przemysłu Kosmetycznego.

[3] Loden M (1992). The increase in skin hydration after application of emollients with different amounts of lipids. Acta Derm Venereol.

[4] van Zuuren EJ, Fedorowicz Z, Christensen R i wsp. (2017). Emollients and moisturisers for eczema. Cochrane Database Syst Rev.

[5] Farage MA, Hood W, Odio M i wsp. (2015). Skin Surface pH and topical emollient: fact or artifact?. JJ Expt Derm Res.

[6] Hon KL, Kung JSC, Ng WGG, Leung TF (2018). Emollient treatment of atopic dermatitis: latest evidence and clinical considerations. Drugs in Context.

[7] Czarnecka-Operacz M, Sadowska-Przytocka A (2015). Pielęgnacja skóry chorego na AZS w świetle nowoczesnej wiedzy medycznej. Alergia.

[8] Rissman R, Oudshoorn MH, Kocks i wsp. (2008). Lanolin-derived lipid mixtures mimic closely the lipid composition and organization of vernix caseosa lipids. Biochim Biophys Acta.

[9] Czarnowicki T, Malajian D, Khattri S i wsp. (2016). Petrolatum: Barrier repair and antimicrobial responses underlying this „inert” moisturizer. J Allergy Clin Immunol.

[10] (2009). EFSA Panel on Food Additives and Nutrient Sources added to Food (ANS). EFSA Journal.

[11] Garty BZ (1986). High plasma urea concentration in babies with lamellar ichtyosis. Arch Dis Child.

[12] Werfel T, Heratizadeh A, Aberer W i wsp. (2016). S2k guideline on diagnosis and treatment of atopic dermatitis – short version. Allergo J Int.

[13] Kerschner M, Williams S, Ring J., Przybilla B., Ruzicka T. (2006). Handbook of Atopic Eczema.

[14] Wollenberg A, Oranje A, Deleuran M i wsp (2015). position paper on diagnosis and treatment of atopic dermatitis in adult and paediatric patients. JEADV 2016.

[15] Manuschiatti W, Maibach HI (1996). Hyaluronic acid and skin: wound healing and aging. Int J Dermatol.

[16] (2017). Cosmetic Ingredient Review Expert Panel Report. Safety assessment of panthenol, panthotenic acid, and derivatives as used in cosmetics. CIR.

[17] Hoare C, Li Wan Po A, Williams H. (2000). Systematic review of treatments for atopic eczema. Health Technol Assess.

[18] Palmer CN, Irvine AD, Terron-Kwiatkowski A i wsp. (2006). Common loss-of-function variants of the epidermal barrier protein filaggrin are a major predisposing factor for atopic dermatitis. Nat Genet.

[19] Elias PM (2014). Lipid abnormalities and lipid-based repair strategies in atopic dermatitis. Biochim Biophys Acta.

[20] Kezic S, Kemperman PM, Koster ES i wsp. (2008). Loss-of-function mutations in the filaggrin gene lead to reduced level of natural moisturizing factor in the stratum corneum. J Invest Dermatol.

[21] Kim BE, Leung DYM (2018). Significance of skin barrier dysfunction in atopic dermatitis. Allergy Asthma Immunol Res.

[22] Kennedy EA, Connolly J, Hourihane JO`B i wsp. (2017). Skin microbiome before development of atopic dermatitis: early colonization with commensal staphylococci at 2 months is associated with a lower risk of atopic dermatitis at 1 year. J Allergy Clin Immunol.

[23] Petry V, Lipnharski C, Bessa GR, Silveira VB, Weber MB (2013). Prevalence of community-acquired methicillin-resistant Staphylococcus aureus and antibiotic resistance in patients with atopic dermatitis in Porto Alegre, Brazil. Intern J Dermatol.

[24] Meylan P, Lang C, Mermoud S i wsp. (2017). Skin colonization by Staphylococcus aureus precedes the clinical diagnosis of atopic dermatitis in infancy. J Invest Dermatol.

[25] Berardesca E, Barbareschi M, Veraldi S, Pimpinelli N (2001). Evaluation of efficacy of a skin lipid mixture in patients with irritant contact dermatitis, allergic contact dermatitis or atopic dermatitis: a multicenter study. Contact Dermatitis.

[26] Loden M, Andersson AC, Andersson C i wsp. (2001). Instrumental and dermatologist evaluation of the effect of glycerine and urea on dry skin in atopic dermatitis. Skin Res Technol.

[27] Loden M, Andersson AC, Anderson C i wsp. (2002). A double-blind study comparing the effect of glycerin and urea on dry, eczematous skin in atopic patients. Acta Derm Venereol.

[28] Amichai B, Grunwald MH (2009). A randomized, double-blind, placebo-controlled study to evaluate the efficacy in AD of liquid soap containing 12% ammonium lactate + 20% urea. Clin Exp Dermatol.

[29] Sugarman JL, Parish LC (2009). Efficacy of a lipid-based barrier repair formulation in moderate-to- severe pediatric atopic dermatitis. J Drugs Dermatol.

[30] Bissonette R, Maari C, Provost N i wsp. (2010). A double-blind study of tolerance and efficacy of a new urea-containing moisturizer in patients with atopic dermatitis. J Cosmet Dermatol.

[31] Draelos ZD (2011). A clinical evaluation of the comparable efficacy of hyaluronic acid-based foam and ceramide-containing emulsion cream in the treatment of mild-to-moderate atopic dermatitis. J Cosmet Dermatol.

[32] Wiren K, Nohlgard C, Nyberg F i wsp. (2009). Treatment with a barrier-strengthening moisturizing cream delays relapse of atopic dermatitis: a prospective and randomized controlled clinical trial. JEADV.

[33] Lucky AW, Leach AD, Laskarzewski P i wsp. (1997). Use of an emollient as a steroid-sparing agent in the treatment of mild to moderate atopic dermatitis in children. Pediatr Dermatol.

[34] Loden M, Andersson AC, Lindberg M (1999). Improvement in skin barrier function in patients with atopic dermatitis after treatment with a moisturizing cream (Canoderm). Br J Dermatol.

[35] Grimalt R, Mengeaud V, Cambazard F, Study Investigators Group. (2007). The steroid-sparing effect of an emollient therapy in infants with atopic dermatitis: a randomized controlled study. Dermatology.

[36] Msika P, De Belilovsky C, Piccardi N i wsp. (2008). New emollient with topical corticosteroid-sparing effect in treatment of childhood atopic dermatitis: SCORAD and quality of life improvement. Pediatr Dermatol.

[37] De Belilovsky C, Roo-Rodriguez E, Baudouin C i wsp. (2011). Natural peroxisome proliferator-activated receptor-alpha agonist cream demonstrates similar therapeutic response to topical steroids in atopic dermatitis. J Dermatolog Treat.

[38] Simpson E, Dutronc Y (2011). A new body moisturizer increases skin hydration and improves atopic dermatitis symptoms among children and adults. J Drugs Dermatol.

[39] Glatz M, Jo JH, Kennedy EA i wsp. (2018). Emollient use alters skin barrier and microbes in infants at risk for developing atopic dermatitis. Plos One.

[40] Simpson EL, Chalmers JR, Hanifin JM i wsp. (2014). Emollient enhancement of the skin barrier from birth offers effective atopic dermatitis prevention. J Allergy Clin Immunol.

[41] Horimukai K, Morita K, Narita M i wsp. (2014). Application of moisturizer to neonates prevents development of atopic dermatitis. J Allergy Clin Immunol.

[42] Darmstadt GL, Ahmed S, Ahmed AS, Saha SK (2014). Mechanism for prevention of infection in preterm neonates by topical emollients: a randomized, controlled clinical trial. Pediatr Infect Dis J.

[43] Belloni G, Pinelli S, Veraldi S (2005). A randomized, double-blind, vehicle-controlled study to evaluate the efficacy and safety of MAS063DP (Atopiclar) in the treatment of mild to moderate atopic dermatitis. Eur J Dermatol.

[44] Abramovits W, Boguniewicz M for the Adult Atopiclar Study Group. (2006). A multicenter, randomized, vehicle-controlled clinical study to examine the efficacy and safety of MAS063DP (Atopiclar) in the management of mild to moderate atopic dermatitis in adults. J Drugs Dermatol.

[45] Boguniewicz M, Zeichner JA, Eichenfield LF i wsp. (2008). MAS063DP is effective monotherapy for mild to moderate atopic dermatitis in infants and children: a multicenter, randomized, vehicle-controlled study. J Pediatr.

[46] Patrizi A, Capitanio B, Neri I i wsp. (2008). A double-blind, randomized, vehicle-controlled clinical study to evaluate the efficacy and safety of MAS063DP (Atopiclar) in the management of atopic dermatitis in paediatric patients. Pediatr Allergy Immunol.

[47] Miller DW, Koch SB, Yentzer BA i wsp. (2011). An over-the-counter moisturizer is a clinically effective as, and more cost-effective than, prescription barrier creams in the treatment of children with mild-to-moderate atopic dermatitis: a randomized, controlled trial. J Drugs Dermatol.

[48] Eichenfield LE, Ahluwalia J, Waldman A i wsp. (2017). Current guidelines for the evaluation and management of atopic dermatitis: A comparison of the Joint Task Force Practice Parameter and American Academy of Dermatology Guidelines. J Allergy Clin Immunol.

[49] (2011). Scottish Intercollegiate Guidelines Network (SIGN. Management of atopic eczema in primary care. SIGN publication no. 125.

